# Medications for psychosis in people with schizophrenia: what happens if you take them and what happens if you don’t?

**DOI:** 10.1017/S1092852925100448

**Published:** 2025-07-25

**Authors:** Ambarin Faizi, Stephen M. Stahl

**Affiliations:** 1 California Department of State Hospitals, Sacramento, CA, USA; 2 Department of Psychiatry, University of California, San Diego, CA, USA

**Keywords:** Schizophrenia, antipsychotic treatment, long-term outcomes, psychopharmacology, duration of untreated psychosis

## Abstract

Schizophrenia is a severe and disabling psychiatric illness that profoundly affects a person’s ability to think clearly, perceive reality, manage emotions, and engage in daily activities. While antipsychotic medications have long been the cornerstone of treatment, debates persist around their long-term use and potential impacts on brain structure and function. In our review, we examine whether antipsychotic medications improve or worsen long-term outcomes in schizophrenia, particularly when treatment is refused or discontinued. Drawing from randomized controlled trials, large-scale observational studies, forensic outcome data, international guidelines, and neuroimaging research, the findings demonstrate that sustained antipsychotic treatment significantly reduces relapse, improves functional outcomes, and may protect against neurobiological deterioration. In contrast, untreated or inconsistently treated psychosis is associated with higher relapse rates, treatment resistance, cognitive decline, and progressive brain changes. While treatment must be personalized and compassionate, the cumulative evidence supports the critical role of early and continuous antipsychotic use in preserving health, autonomy, and long-term recovery for individuals living with schizophrenia.

## Introduction

Schizophrenia is a severe and chronic psychiatric illness that disrupts perception, cognition, emotion, and function. Despite its low prevalence, approximately 1% globally, it remains one of the leading causes of disability worldwide.^1^ While historically viewed as a progressively debilitating mental illness, the disorder has undergone a paradigm shift in understanding. Once associated with treatment nihilism, contemporary research underscores that with timely and appropriate interventions, individuals diagnosed with schizophrenia can achieve significant symptom remission and functional recovery.^2^ ^–^^5^

Antipsychotic medications, the cornerstone of schizophrenia treatment for over 70 years, have demonstrable efficacy in reducing psychotic symptoms, preventing relapse, and improving functional outcomes. Yet, the role of long-term pharmacotherapy remains controversial. A vocal minority of critics argue that antipsychotics impair brain function, blunt emotions, and are overprescribed, pointing to naturalistic studies suggesting better outcomes in unmedicated patients. This controversy has led to an urgent clinical and ethical question: Do antipsychotic medications improve long-term outcomes in schizophrenia or can they worsen them? This question becomes even more pressing when patients intermittently refuse treatment, a common occurrence in clinical practice largely due to anosognosia (poor insight), an unfortunate and prominent symptom of schizophrenia. As clinicians, we must weigh the potential neurobiological effects of both antipsychotic treatment and untreated psychosis. The stakes are high: untreated schizophrenia is associated with persistent symptoms, cognitive decline, impaired functioning, poor quality of life, and even premature death.

Our aim is to evaluate the efficacy and necessity of antipsychotic medications in the treatment of schizophrenia, particularly in light of the adverse outcomes observed in patients who intermittently refuse or discontinue treatment. First, we review short-term randomized controlled trials (RCTs), long-term naturalistic studies, real-world forensic outcomes, and emerging neuroimaging evidence to determine whether sustained antipsychotic treatment offers a net benefit in reducing relapse, preserving function, and improving clinical outcomes. We then discuss what we know about the consequences of not taking medications for psychosis in people with schizophrenia, including putative neurotoxic and functional consequences of untreated psychosis.

## What happens if people with schizophrenia take antipsychotic medications for psychosis?

### Short-term trials

Short-term RCTs form the evidentiary backbone of antipsychotic medication approval. These studies typically last 4–6 weeks and assess symptom reduction using validated scales like the Positive and Negative Syndrome Scale (PANSS) or Brief Psychiatric Rating Scale (BPRS). These trials consistently show that a 20% reduction in symptom severity can be observed within the first 2–4 weeks of treatment. For example, in studies^6^ evaluating antipsychotic medications such as risperidone and olanzapine, clinically meaningful improvements typically occur within this initial treatment window. This has led to the understanding that the first few weeks of treatment are critical for assessing early response and predicting longer-term outcomes. Short-term RCTs remain the gold standard for demonstrating the initial efficacy of antipsychotic medications. They consistently show that antipsychotics significantly reduce positive symptoms of schizophrenia, such as delusions and hallucinations. Despite their brevity, their findings are consistent and robust: antipsychotic medications are significantly more effective than placebo in reducing psychotic symptoms and preventing relapse.^5^ ^,^^7^ ^–^^12^

In one of the most comprehensive meta-analyses of antipsychotic efficacy, Leucht et al.^10^ reviewed 167 double-blind RCTs, spanning 60 years of antipsychotic drug trials in acute schizophrenia and with over 28,000 chronically ill participants, and clarified what constitutes a clinically meaningful treatment response. While regulatory trials often define “response” as a 20% reduction in symptom severity on rating scales such as the PANSS or BPRS, this minimal threshold reflects modest clinical improvement. A 20% reduction may signify early symptom shift, but it does not reliably correlate with improved functioning or reduced suffering. Notably, Leucht and colleagues differentiated this from “good response,” defined as a 50% or greater reduction in symptoms scores: a benchmark more strongly associated with robust clinical change, patient-perceived benefit, and readiness for social recovery. Their meta-analysis found that patients treated with antipsychotic medications were more likely to achieve this level of improvement compared to those receiving placebo. These findings strengthen the argument that antipsychotics do more than merely nudge symptoms—they enable substantial and clinically visible improvement in a population where early and effective treatment can alter long-term trajectories. The findings further reinforce the importance of distinguishing between minimal and meaningful response when evaluating the utility of antipsychotic medications in short-term trials.

In summary, short-term trials robustly support the initial efficacy of treatment with antipsychotic medications.

### Long-term trials

Despite the proven efficacy of antipsychotics in short-term trials in people with schizophrenia, a critical question remains: do these benefits persist in the long term? While short-term RCTs provide critical initial evidence for the efficacy of antipsychotic medication, particularly in reducing acute psychotic symptoms, these trials are inherently limited by their brief duration. In the context of a chronic and relapsing condition like schizophrenia, longer-term outcomes such as sustained remission, functional recovery, and prevention of relapse are more clinically meaningful. However, conducting long-term RCTs in this population presents ethical and practical challenges. Randomizing individuals to prolonged placebo conditions or medication discontinuation is widely considered unethical due to the known risk of relapse and potential for serious harm to the individual and others. Furthermore, the chronic nature of schizophrenia, often accompanied by fluctuating insight, impaired adherence, and social instability, makes participant retention in blinded long-term trials difficult. These limitations underscore the importance of real-world data, observational studies, and large registry-based analyses to examine the long-term effectiveness and safety of antipsychotic treatment in naturalistic settings.

The large-scale, Schizophrenia Outpatient Health Outcomes (SOHO) study^13^ provides support for the long-term effectiveness of antipsychotic medications in real-world settings. Encompassing over 10,000 patients across 10 European countries, SOHO is one of the largest longitudinal studies ever conducted on the outpatient treatment of schizophrenia. The authors observed patients over a 3-year period to assess symptom severity, functional outcomes, and treatment adherence under naturalistic clinical conditions. Importantly, the study reflected the diversity of schizophrenia treatment outside of controlled research environments, offering insights that are directly applicable to everyday psychiatric practice. The findings were unequivocal in supporting the sustained use of antipsychotics. Patients who remained adherent to treatment, particularly with olanzapine and clozapine, demonstrated greater improvements in clinical outcomes, including reductions in positive and negative symptoms, and improvement in quality of life and social functioning, reinforcing the idea that long-term pharmacologic engagement translates to tangible benefits in patient quality of life. Similar to the CATIE trial,^12^ the researchers found olanzapine to be more effective than other antipsychotic medications in the same class. The 6-month results indicating better clinical outcomes including quality of life and symptom improvement measured with the Clinical Global Impressions (CGI) scale were also sustained in the 12-month analysis of participants treated with olanzapine and clozapine. The results also confirmed greater effectiveness of clozapine and supported using combinations of antipsychotics.

Another compelling real-world study^14^ offers validation of antipsychotic efficacy beyond short-term RCTs. This study examined outcomes among individuals with serious mental illness, including schizophrenia, who were deemed incompetent to stand trial (IST) and required psychiatric stabilization and competency restoration in a forensic setting. The authors found that among 3166 individuals deemed as IST, 86.5% were successfully restored to competency within 1 year of antipsychotic treatment. This study highlights both the clinical effectiveness of antipsychotic medications as well as their ability to restore high-level capacities and function in severely mentally ill populations.

Further affirming their central role in the treatment of schizophrenia, Glick, Correll, and colleagues^15^ offer a comprehensive, data-driven synthesis of the mid- and long-term efficacy of antipsychotic medications. Drawing from extensive evidence of studies with a duration of 3 months or longer, the authors concluded that their findings from controlled and observational long-term studies “closely parallels the efficacy observed in short-term controlled studies.” Glick and colleagues provided treatment recommendations in support of long-term treatment of schizophrenia. Based on their findings, the literature, and clinical experiences, they emphasized that antipsychotics consistently reduce relapse risk, improve symptom stability, and enhance functional outcomes over the long term, especially when used in a personalized and continuous manner. A personalized model underscores the need to optimize the use of antipsychotics across the illness trajectory and strengthens the agreement that, when applied thoughtfully, antipsychotic medications confer not only short-term relief but also durable, life-stabilizing benefits in patients with schizophrenia.

Additional support for long-term antipsychotic treatment comes from the systematic review conducted by Takeuchi, Suzuku, Uchida, et al.^16^ which examined 14 international clinical practice guidelines and pharmacological treatment algorithms for schizophrenia published after the year 2000. Their review demonstrated a remarkable global consensus: virtually all authoritative guidelines recommend continued antipsychotic maintenance therapy for patients stabilized after an acute episode to prevent relapse. The authors noted that while the exact duration of maintenance therapy may be up for debate, no major guideline supports discontinuation within 5 years of illness onset. Furthermore, intermittent or targeted strategies, defined by the National Institute for Health and Clinical Excellence Core^17^ (NICE) as “the use of antipsychotic medication only during periods of incipient relapse or symptom exacerbation rather than continuously,” also were not recommended. Takeuchi’s comprehensive synthesis affirms that long-term pharmacotherapy is not only evidence-based but also internationally endorsed as a standard of care, especially when tailored to individual risk profiles, functional status, and patient preferences.

Additional evidence for the value of long-term treatment of people with schizophrenia with antipsychotics comes from the largest real-world cohort studies examining schizophrenia outcomes by Tiihonen et al.^18^ ^,^^19^ and Taipale et al.^20^ These studies illustrate that continuous antipsychotic use is associated with reduced relapse rates, lowered mortality, and improved long-term functioning. Specifically, in a nationwide cohort of 29,823 patients with schizophrenia in Finland, Tiihonen and colleagues compared the effectiveness of antipsychotic treatment strategies using observational data and within-individual analyses. The results demonstrated that continuous antipsychotic use was associated with a dramatically reduced risk of hospitalization and all-cause mortality. In particular, this study found that clozapine and long-acting injectables (LAIs) were the most effective in preventing hospitalization, with clozapine associated with a 22% lower risk of rehospitalization compared to olanzapine, and LAIs significantly outperforming their oral counterparts. In the complementary FIN20 study by Taipale et al., a nationwide longitudinal analysis of over 62,000 patients with schizophrenia and up to two decades of follow-up (median 14.1 years), researchers found that long-term antipsychotic use conferred a substantial survival benefit. Compared to no antipsychotic use, “long-term antipsychotic use was associated with substantially lower all-cause, cardiovascular and suicide mortality in people with schizophrenia.” Furthermore, in the 20-year nationwide follow-up study,^19^ the authors cautioned that the “risk of treatment failure or relapse after discontinuation of antipsychotic use does not decrease during the first 8 years of illness, and that long-term antipsychotic treatment is associated with increased survival.” These high-quality, population-level studies provide some of the most convincing evidence that continuous antipsychotic treatment not only prevents relapse but saves lives.

Collectively, these studies illustrate that long-term antipsychotic treatment is associated with a substantial reduction in hospitalization rates and reduced risk of death from all causes, including cardiovascular disease and suicide, among individuals with schizophrenia.

## What happens if people with schizophrenia do not take antipsychotic medications for psychosis?

### No harm, no foul?

Although antipsychotic medications have demonstrated efficacy in short- and long-term trials in people with schizophrenia, their long-term impact remains a subject of ongoing inquiry: for whom do these benefits persist in the long run, and what happens when patients discontinue treatment or go untreated? Some patient advocates argue that if the short-term trials show only about a 20% reduction in positive symptoms for responders, with minimal improvement in negative symptoms, quality of life, and social functioning, and that if not all patients even achieve this modest benefit, then antipsychotic treatment might reasonably be considered optional. Their position is that a patient could always resume medication later and potentially obtain the same symptom relief if they change their mind. They point to evidence that a portion of patients recover without sustained antipsychotic treatment, citing studies that report recovery rates around 21% without treatment in first-episode schizophrenia^21^ and roughly 11% in patients with multi-episode schizophrenia,^22^ suggesting that the cost and side effects of treatment might not be justified for everyone if there is the chance to recover without it. For example, Harrow and Jobe’s observational studies^23^ ^,^^24^ demonstrated that a subgroup of individuals with schizophrenia who discontinued antipsychotic medication were less likely to be psychotic and experience more periods of recovery compared to their continuously medicated counterparts. Similarly, Wunderink et al.^25^ found that among first-episode patients in remission, dose reduction or discontinuation was associated with improved long-term social recovery at the 7-year mark. Such findings are often cited in debates questioning the need for long-term pharmacologic maintenance. On the other hand, critics of these studies have raised important counterpoints, notably on the study design, patient selection, and illness severity. They highlight that patients who discontinue medication successfully may represent a fundamentally different clinical phenotype, such as those with a milder course of illness and an intrinsically better prognosis or those with greater psychosocial resilience, rather than evidence that medication itself causes poor outcomes. Supporting this, a study more closely generalizable to real-world settings by Robinson et al.^26^ prospectively followed patients after their first episode of schizophrenia or schizoaffective disorder and found that patients who discontinued antipsychotic medication had a 5-fold higher risk of relapse compared to those who maintained treatment. In their study, 53% of patients who relapsed for the first time after 2 years of stability had discontinued medication use, “suggesting the continued importance of maintenance medication treatment” extending beyond the vulnerable early years following illness onset.

### The brain pays the price of relapse

There has been a growing discussion around the impact of intermittent or targeted treatment in patients with partial insight and adherence difficulties and even some proposals to curtail continuous treatment. However, numerous studies show that each psychotic relapse is neurobiologically costly, contributes to treatment resistance, and that the brain may not fully recover to baseline functioning after repeated episodes.^2^ Emsley et al.^27^ emphasized that the factor most strongly correlated with brain volume decline is relapse duration, reinforcing the idea that the chronicity of untreated psychosis is neurotoxic. While individualized treatment planning may involve dose reductions or carefully supervised discontinuation in rare cases, unstructured or prolonged refusal of medication should not be misconstrued as benign. The long-term risks, including symptom exacerbation, treatment resistance, suicidality, and functional decline, highlight the need for proactive monitoring and ongoing patient engagement, even after symptom remission. A more realistic and compassionate harm-reduction model may involve minimizing relapse duration, using LAIs when appropriate, and maintaining the lowest effective dose tailored to individual needs. Taken together, these findings affirm that while schizophrenia is a heterogeneous and complex illness, the vast majority of patients derive significant benefit from sustained pharmacologic treatment. Rather than reject maintenance treatment altogether, these studies underscore the importance of tailoring care to the patient’s clinical trajectory, risk profile, and preferences, using shared decision-making grounded in the best available evidence including assessment of the risk of long-term progressive brain damage worsened by lack of continuous treatment.

As schizophrenia is now known to be associated with structural brain abnormalities, including reductions in gray matter volume, particularly in the prefrontal cortex, hippocampus, and temporal lobes,^28^ ^,^^29^ a critical debate in the literature concerns whether long-term structural brain changes, especially reductions in gray matter volume, are due to illness or to the treatment. A close examination of the literature reveals a complex and yet reassuring picture. The available evidence increasingly supports the view that schizophrenia itself is associated with progressive neuroanatomical deterioration and that this disease-driven brain toxicity may be mitigated, not worsened, by antipsychotic treatment. Concerns about potential brain volume loss due to antipsychotic medications persist, but these concerns are increasingly counterbalanced by growing literature suggesting that schizophrenia itself is far more neurotoxic than its treatment. An extensive review by Fountoulakis and Stahl^29^ reveals that while some brain changes may be observed in medicated patients, these are often less pronounced than changes seen in those who remain untreated and are more plausibly linked to illness progression.^29^ ^,^^30^ Fountoulakis & Stahl^29^ argue that the literature does not support that antipsychotics cause loss of brain volume, rather that they might exert a protective effect against brain volume loss. Lawrie^31^ additionally dismantled unfounded fears about antipsychotics, stating they “probably do not shrink the brain” and that this does not have clinical relevance.

More recently, a landmark triple-blind randomized, placebo-controlled MRI study by Chopra et al.^30^ further clarified this issue, showing that patients treated with second-generation antipsychotics exhibited less progression of gray matter loss over time, and in some, even reversal of volume deficits. They concluded that antipsychotic medication may possibly reverse illness-related volume loss in the pallidum and noted that their findings were consistent with earlier studies that suggest a neuroprotective effect of second-generation antipsychotic medication. This aligns with the findings from an analysis of 202 patients with MRI data from the Iowa Longitudinal Study of first-episode schizophrenia, in which Emsley^27^ found that relapse duration (not the number of relapses) and treatment intensity are associated with “significant reductions in brain volume measures.” Although continuous treatment remains ideal for symptom control and relapse prevention, these findings suggest that any antipsychotic exposure, whether consistent or sporadic, may confer neuroprotective benefits at appropriate dosages, preferably at the lowest medication dosage indicated. This paradigm offers hope for patients with adherence challenges, reframing discontinuous treatment as a viable harm-reduction strategy.

### The cost to the brain of the delay in the onset of treatment

Perhaps the most convincing evidence that schizophrenia is associated with progressive structural brain degeneration comes from studies of how long after the onset of a first psychotic episode that treatment is delayed. Emerging evidence from neuroimaging, clinical outcomes, and biomarker studies reinforces a critical principle: in schizophrenia, as in stroke or traumatic brain injury, “time is brain.” The Duration of Untreated Psychosis (DUP), defined as the interval between the onset of psychotic symptoms and the initiation of effective antipsychotic treatment, has been consistently associated with poorer long-term outcomes, reduced functional recovery, and progressive structural brain changes.^3^ ^,^^32^ ^,^^33^ In the United States, Kane et al.^34^ reported that the average DUP among first-episode patients was 74 weeks, well beyond the under 12-week target recommended by the World Health Organization and the International Early Psychosis Association. This prolonged delay represents a critical window of missed opportunity, during which patients are often deteriorating in the absence adequate treatment.

Neuroimaging studies have shown that even modest delays in treatment contribute to reductions in gray matter volume, most pronounced in the prefrontal cortex, hippocampus, and temporal lobes^28^ ^,^^29^ regions responsible for memory, executive functioning, and emotional regulation. Poor outcomes are associated with prominent hippocampal volume reduction in schizophrenia and with long DUP.^28^ These changes may be evident even before the transition to full-blown psychosis, suggesting that neurobiological alterations occur in the prodromal phase.^35^ Prolonged DUP has been associated with hippocampal atrophy and decreased gray matter volume in orbital-frontal, parietal,^36^and occipitotemporal regions,^37^ reinforcing the hypothesis that untreated psychosis may have neurotoxic effects on several brain structures essential for memory and executive function. Guo et al.^37^ aligned with Goff^28^ suggesting that untreated psychosis itself drives structural brain changes and that structural brain abnormalities are associated with poor treatment response and poor long-term outcomes, thereby, stressing the importance of early detection and treatment. Stone et al.^33^ proposed a dual pathway, where both neurodevelopmental vulnerabilities and neurodegenerative processes contribute to cognitive deficits in long-term untreated schizophrenia. In 2020, Kraguljac et al.^38^ added to this body of work with a multimodal imaging study showing that first-episode patients already exhibit widespread abnormalities in brain structure, particularly white matter integrity and glutamatergic metabolism at the time of diagnosis. A major strength of their study was that they recruited participants who were either antipsychotic medication-naïve (83%) or had less than or equal to 5 days of antipsychotic exposure in their lifetime. This contributes to efforts in disentangling skepticism related to antipsychotic medication effects from inherent illness deterioration. Furthermore, their findings indicate that “DUP exerts its effects on treatment response through affecting white matter integrity,” adding to a growing body of evidence of white matter integrity as a predictor of treatment response and echoing the importance of early intervention efforts in schizophrenia.

These findings emphasize the urgent need for early identification and intervention in schizophrenia. The longer a patient remains untreated, the more likely they are to experience neuroanatomic deterioration, functional decline, and treatment resistance. Studies^32^ show that even short delays of 7 days reduce functional recovery, with the steepest decline occurring within the first month. Recent research by Slovakova et al.^39^ added nuance: differences as slight as weeks in DUP substantially translated to worse outcomes 5 years later. For patients with adherence challenges, evidence increasingly supports harm-reduction strategies, such as low-dose maintenance, mostly given that relapse duration, not number of relapses, correlates most strongly with brain volume loss.^27^

## Timely intervention, lasting recovery: advancing evidence-based treatment for schizophrenia

The findings we presented throughout this review converge on a central message: the timely and sustained use of antipsychotic medication remains the most effective evidence-based strategy for reducing symptoms, preventing relapse, and optimizing functional outcomes in schizophrenia. While concerns about potential side effects and long-term safety have fueled debate, these must be balanced against the substantial and well-documented risks associated with untreated or inconsistently treated psychosis. It is a myth that there are no consequences to refusing treatment of psychosis with antipsychotics in schizophrenia and that reinstituting treatment at a later date will always work as well as if it had been continuously given.

Rather than a one-size-fits-all approach (ie, everyone with schizophrenia needs to be treated continuously forever versus everyone with schizophrenia can stop their medications with impunity), contemporary care must reflect the heterogeneity of schizophrenia. Individuals vary in their symptom trajectories, functional goals, treatment tolerability and response, and levels of insight. The goal is not to promote indiscriminate medication use but rather to emphasize early initiation and thoughtful, individualized treatment as the standard, particularly in light of data showing that treatment delays and interruptions are associated with poorer outcomes. Thus, the all-too-common situation of intermittent or inconsistent treatment remains a serious clinical risk. While harm-reduction approaches may provide flexibility and reduce barriers for some, the cumulative burden of relapse, particularly prolonged episodes, underscores the value of sustained therapeutic engagement. The evidence increasingly supports that every episode of untreated psychosis matters. The longer psychosis is left untreated, the more difficult it becomes to reverse its impact. Moreover, neuroimaging findings and real-world outcomes suggest that untreated schizophrenia is more neurotoxic than appropriately dosed antipsychotic medications. Although ongoing refinement in treatment algorithms and novel medications may be necessary, the pendulum should not swing so far toward skepticism that it obscures reality: antipsychotic medications remain the cornerstone of recovery for individuals with schizophrenia.

In reframing this conversation, we must promote a nuanced narrative: one that upholds patient autonomy while also acknowledging the realities of a prominent symptom of schizophrenia, lack of insight into the illness. As the field moves forward, integration of biological, clinical, and lived-experience approaches will be essential to inform shared decision-making and develop more personalized, ethical, compassionate, and effective care strategies for people living with schizophrenia.
